# Design of Experiments to Study the Impact of Process Parameters on Droplet Size and Development of Non-Invasive Imaging Techniques in Tablet Coating

**DOI:** 10.1371/journal.pone.0157267

**Published:** 2016-08-22

**Authors:** Thomas J. Dennison, Julian Smith, Michael P. Hofmann, Charlotte E. Bland, Raj K. Badhan, Ali Al-Khattawi, Afzal R. Mohammed

**Affiliations:** 1 Aston School of Pharmacy, Aston University, Birmingham, United Kingdom; 2 Viridian Pharma Ltd, Newport, United Kingdom; 3 Biomaterials Unit, School of Dentistry, University of Birmingham, Birmingham, United Kingdom; 4 Aston Research Centre for Healthy Ageing, Aston University, Birmingham, United Kingdom; Taipei Medical University, TAIWAN

## Abstract

Atomisation of an aqueous solution for tablet film coating is a complex process with multiple factors determining droplet formation and properties. The importance of droplet size for an efficient process and a high quality final product has been noted in the literature, with smaller droplets reported to produce smoother, more homogenous coatings whilst simultaneously avoiding the risk of damage through over-wetting of the tablet core. In this work the effect of droplet size on tablet film coat characteristics was investigated using X-ray microcomputed tomography (XμCT) and confocal laser scanning microscopy (CLSM). A quality by design approach utilising design of experiments (DOE) was used to optimise the conditions necessary for production of droplets at a small (20 μm) and large (70 μm) droplet size. Droplet size distribution was measured using real-time laser diffraction and the volume median diameter taken as a response. DOE yielded information on the relationship three critical process parameters: pump rate, atomisation pressure and coating-polymer concentration, had upon droplet size. The model generated was robust, scoring highly for model fit (R^2^ = 0.977), predictability (Q^2^ = 0.837), validity and reproducibility. Modelling confirmed that all parameters had either a linear or quadratic effect on droplet size and revealed an interaction between pump rate and atomisation pressure. Fluidised bed coating of tablet cores was performed with either small or large droplets followed by CLSM and XμCT imaging. Addition of commonly used contrast materials to the coating solution improved visualisation of the coating by XμCT, showing the coat as a discrete section of the overall tablet. Imaging provided qualitative and quantitative evidence revealing that smaller droplets formed thinner, more uniform and less porous film coats.

## Introduction

The atomisation of a liquid stream into a fine spray is utilised in a variety of industries for a range of applications, including the pharmaceutical industry, where it is most notably employed for tablet film coating [1,2]. During the atomisation process individual droplets evolve from a liquid stream with a concurrent increase in surface area to mass ratio of the liquid [3]. In principle, for atomisation to occur it is necessary to generate a high relative velocity between the liquid stream and the surrounding air or gas [4,5]. The ratio of the flow rates of the atomising air and the liquid stream, known as the atomisation to liquid flow ratio (ALM), is considered an important parameter in determining droplet size [6,7]. Various reports highlight the dependency of droplet size on both atomisation pressure and liquid flow rate (pump rate) [8,9] and both have been recognised as critical process parameters (CPPs) for coating processes [10]. Viscosity is also known to significantly influence droplet size during atomisation [11,12], with higher viscosity liquids forming larger droplets during atomisation.

Twin-fluid atomisation is a complex and multivariable process that, despite significant efforts, is still not well understood and remains difficult to predict [11]. The general agreed mechanism involves an initial sheet formation of the liquid stream after exiting the nozzle, followed by a breakup into stretched liquid ligaments and then droplet formation [1,13–15]. A more recent and complex two-stage instability mechanism comprises the formation of an initial shear instability forming waves on the liquid surface and then a Raleigh-Taylor instability at the wave crests, forming ligaments that stretch and further break up into droplets [11,16–18]. Unfortunately for tablet film coating, many studies have not been undertaken using non-Newtonian (viscous) fluids [19].

Production of a high quality tablet film coat depends upon multiple factors such as the formulation [20], equipment [21] and process parameters [22,23]. The importance of droplet size on coating efficiency and quality has been reported, [14,24,25] with small droplets responsible for a more homogenous or even distribution of coating solution on the tablet surface [26,27]. More rapid water evaporation due to the greater volume to surface area ratio of small droplets [28–30] leads to greater coating efficiency [31]. Over-wetting, which can result in defects such as poor adhesion of coating polymer to the tablet surface, peeling, twinning, picking and sticking and tablet erosion [2,32], is thus less prevalent with smaller droplets. Tablet defects, particularly poor adhesion can harm film functionality and negatively impact on the mechanical properties provided by a film coat. Typical droplet sizes in fluidised bed coating range between 20 and 100 μm, with coating efficiency reportedly being optimal below 10 μm, although this may run the risk of spray-drying of droplets before they reach the tablet surface [31,33]. To date no studies have investigated the direct impact of droplet size on tablet film coat using micro scale imaging. Revealing the micro scale morphology of the coat in this way could provide information on coat quality as well as the interaction of the coat with the tablet core.

The aim of this study was to investigate and identify the differences in tablet film coats produced from either small or large droplets using micro imaging techniques. To produce droplets of a known size a design of experiments (DOE) approach was implemented to evaluate the impact of three CPPs: atomisation pressure, pump rate and polymer concentration on droplet size during atomisation of a film coat solution from a twin-fluid external mixing nozzle. The generated model was then exploited to reveal the process conditions required to achieve droplets of a desired size. The hypothesis that small droplets would create films that were more homogenous and concise was then tested non-invasively using confocal microscopy (CLSM) and X-ray microcomputed tomography (XμCT).. CLSM has been used previously for imaging of film coatings [34–36] and XμCT has been used to study tablet microstructure [37], particle coating [38] and tablet coat visualisation [34,39], although common radiopacifying agents to improve contrast have not previously been included. Both imaging techniques provided qualitative and quantitative information that revealed differences in coat characteristics depending on the droplet size used.

## Materials and Methods

### Materials

D-mannitol, D-sorbitol, magnesium stearate, bismuth(III) oxide and barium sulphate were purchased from Sigma–Aldrich (Poole, UK). Polyplasdone XL-10 (crospovidone) was obtained from ISP (Switzerland). Avicel PH102 (MCC) was obtained from FMC Biopolymer (Philadelphia, USA). Aerosil 200 Pharma (colloidal silicon dioxide) was obtained from Evonik Industries (Essen, Germany). A coating polymer Kollicoat IR (BASF, Germany) and a fluorescent dye riboflavin 5′-monophosphate sodium salt (Sigma-Aldrich, Pool, UK) were obtained for film coating work.

### Viscosity Measurements

Viscosity measurements of Kollicoat IR solutions were performed on a Brookfield LVDV-I+ viscometer (Massachusetts, USA) using spindle 1 (for concentrations of 12.5% w/w and below) and spindle 2 (for 20% w/w) at 100rpm, 25°C.

### Tablet Formation

A formulation consisting of MCC (47% w/w), mannitol (23.5% w/w), sorbitol (23.5% w/w), crospovidone (4% w/w) and silicon dioxide (1% w/w) was blended for 5 minutes followed by addition of magnesium stearate (1% w/w) and further blending for 1 minute. Direct compression of tablets (500 mg) at a compaction force of 30 kN and 6 second dwell time was performed using an Atlas T8 automatic press SPECAC^®^ (Slough, UK). A 13mm round, flat faced die was used for tablet production. All tablets were produced under ambient conditions.

### Film Coating and Apparatus

Suspensions of Kollicoat IR (BASF. Germany) were prepared using ultrapure water. The suspensions were pumped and atomized using a Mini Coater Drier-2 (Caleva Process Solutions Ltd., Dorset, UK) comprising a 1/8 JJAU-SS air-actuated external mixing atomising nozzle (Spraying Systems Co., Wheaton, IL, USA). Film coating conditions were determined from the results obtained from the DOE study to obtain desired droplet sizes. In all cases, fluidization air was provided at a velocity of 16 m/s and a temperature of 60°C. Assuming a linear correlation between coating time and film coat thickness for solutions of the same polymer concentration, large droplet coating was performed for 2.5x longer to achieve a similar coating thickness between the two droplet sizes.

### Droplet Size Analysis

Real-time measurement techniques offer the advantage of measuring droplet size ranges and droplet dimensions more accurately [34,40]. Real-time droplet size measurements using laser diffraction was performed on a Spraytec System (Malvern Instruments Ltd, Malvern, UK), to record droplet size distribution under different conditions. In order for the laser to access the spray path the fluidisation chamber was removed and the spray gun was placed 8 cm above the path of the laser beam. The measuring distance to the nozzle was set at 8.5 cm. Each sample was measured in a continuous mode for one minute, with particle size distribution measured once per second. Kollicoat IR solutions were used for droplet size analysis.

## Design of Experiments (DOE)

### CQA and CPP Selection

Critical quality attribute (CQA) and CPP selections were based on reports from the literature concerning the importance of droplet size on coating quality and parameters effecting droplet size during atomisation, discussed earlier. CPP selection was also determined by the limitations of the experimental setup, namely removal of the coating chamber. Droplet volume median diameter (VMD) was selected as a CQA and a range of 20–100 μm chosen based on typical droplet size range during coating and the risk of spray drying at lower droplet sizes. Pump rate, atomisation pressure and viscosity/polymer concentration were chosen as CPPs. Appropriate CPP ranges were founded on the equipment ranges and preliminary work with the apparatus and coating polymer. The atomisation pressure range was set at 1–2 bar and pump rate at 1–4 rpm (corresponding to a flow rate of 10–40 ml/hour). Kollicoat IR concentrations (w/w) were set at 5%, 12.5% and 20% corresponding to a viscosity of 0.99, 3.10 and 15.00 mPa.s respectively.

### Experimental Design

Modelling of the atomisation process was performed using MODDE 10 software (Umetrics, Sweden). A quadratic process model using response surface modelling optimisation with a central composite face-centred design was chosen. This required 17 runs, including 3 centre points. These ranges were used to set low, medium and high levels for each parameter, see Table 1. Medium levels were used for the centre point measurements and were run in triplicate.

**Table 1:** Low, medium and high levels for CPPs. The medium level for each CPP was used for centre point measurements.

	Low	Medium	High
**Pump Rate (rpm)**	1	2.5	4
**Atomisation Pressure (bar)**	1	1.5	2
**Kollicoat IR Concentration (% w/w)**	5	12.5	20

### Confocal Scanning Laser Microscopy (CLSM)

Confocal microscopy was carried out on a CLSM TCS SP5 II System (Leica Microsystems GMBH, UK) using a 10x dry objective. Riboflavin monophosphate sodium was used as a fluorescent dye (0.5% w/w) in the film coat solution, as described by Ruotsalainen et al. [34] and scanned at a wavelength of 458 nm. Maximum projection images were used to analyse the surface morphology based on the intensity of the fluorescence of pixels within each plane. Maximum projection images were also rotated to provide a side view of the film coating to reveal film coat thickness, the morphology of the outer coating surface and also the tablet-core interface.

### X-Ray Microcomputed Tomography (XμCT)

XμCT was performed using a Skyscan 1172 high- resolution micro-CT (Bruker, Belgium). Samples were placed in a Perspex tube and separated by polystyrene spacers. Samples were scanned using an Al/Cu filter, at a pixel size of 6.79 μm, a source voltage of 89 kV, current of 112 μÅ and rotated through 360°at increments of 0.64°. Projections were reconstructed using NRecon software (Skyscan, Version 1.5.11) to produce non-invasive cross-sections of the tablets at sequential z planes.

### Film Coat Water Content

Film coat sections (around 5 mg) were analysed for water content by thermogravimetric analysis (TGA). A PerkinElmer Pyris 1 TGA (Massachusetts, USA) was used to heat samples from 50–150°C (holding for 5 min at 100°C) and % weight loss measured as film coat water content.

### Image Analysis

Porosity measurements of XμCT reconstructions were performed using two separate methods. Bruker-MicroCT CT-Analyser (CTAn, Bruker, Belgium) was used to provide porosity measurements of the coating using the 5 outermost reconstructions in the z-plane, calculating porosity using the porosity plug-in. ImageJ (National Institutes of Health, USA) was used to process the reconstructions by adjusting the image threshold by applying the Huang threshold and subsequent binarisation, followed by measuring the porous area fraction at a set ROI of the coating. Fluorescent coat porosity was measured in the same way as XμCT using the ImageJ method. Film coat thickness at the top and bottom tablet surface for fluorescent coats was performed using ImageJ, starting with image processing through initial contrast adjustment, followed by binarisation, hole filling and despeckling to produce one complete binary section. The local thickness plugin for ImageJ, based upon the algorithm developed by Hildebrand and Rüegsegger [41], was used to measure film coat thickness; this involves fitting spheres within the binary layer and the film coat thickness at any point measured as the diameter of the largest sphere at that point. Surface roughness of the coat was represented by the root mean square (RMS) of the valleys and peaks of the coating, otherwise put as the standard deviation in individual film coat thickness values [42]. Heat maps were generated using the HeatMap From Stack ImageJ plugin by Samuel Péan [43].

## Results and Discussion

A model of the atomisation process was generated from the droplet size data. Model optimisation revealed the parameters required to produce droplets of a given size and this informed the choice of process conditions for tablet coating. Before coating, however, the model required verification and validation.

### DOE

#### Model verification and validation

**Fig 1:**
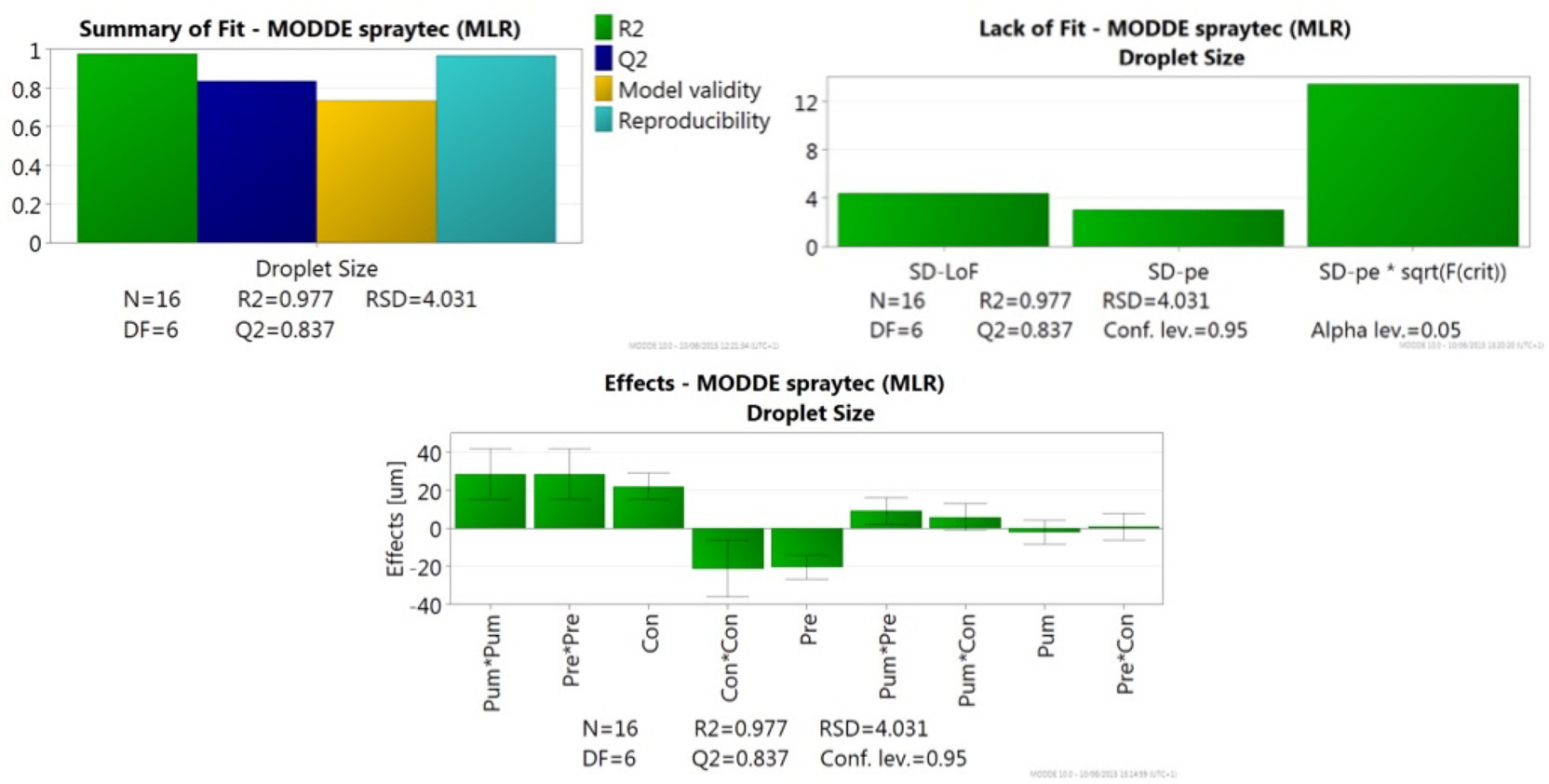
Summary of fit plot showing model fit (R^2^), predictability (Q^2^), model validity and reproducibility. The model has been fitted using RSM. Lack of Fit plot showing standard deviation (SD) due to lack of fit (SD-LoF), SD of pure error (SD-pe) and SD of pure error * the critical F-value (SD-pePsqrt(F(crit)). Effects plot for the three factors: pump rate (Pum), concentration of Kollicoat IR suspension (Conc) and atomisation pressure (Pre). Factors are ordered in terms of impact on droplet size. Confidence interval bars are included for each factor.

A residuals normality plot was used to identify any outliers, resulting in the exclusion of one of the data points from the total 17. The quadratic model generated was fitted against the data and the response is shown in the summary of fit plot (Fig 1), which provides information on the strength and robustness of the model. The R^2^ value of 0.977 signified a low variation in the response (droplet size) and strong fit between the data and the model. The Q^2^ value of 0.837, ideally >0.5, demonstrated a high predictive power, allowing for confident prediction of the effect of changing process parameters on droplet size and process optimisation. The model also demonstrated a strong score for validity of 0.736, far exceeding the required value of >0.25. Similarly the value obtained for reproducibility of 0.967 significantly surpassed the requisite value of 0.5, indicating good experimental control and low pure error.

For further model validation a lack of fit plot and ANOVA were employed to compare the model error and pure error. In the lack of fit plot (Fig 1) the first bar shows standard deviation (SD) due to lack of fit or model error (SD-LoF) and the second bar shows the SD of the pure error (SD-pe). The final bar shows the SD of pure error * the critical F-value (SD-pePsqrt(F(crit)), at the p = 0.05 level of significance. The SD-LoF is much lower than SD-pePsqrt(F(crit), indicating a good fit. The ANOVA shows a very low variance of P <0.00001 due to the regression model, whereas the variance due to residuals and replicate errors was insignificant at a P value of 0.348. The results obtained for both lack of fit and ANOVA validate the model by demonstrating low error due to the model and a low level of pure error in the experimental setup, indicating good control over the experiment.

#### Regression model equations and factor effects

The regression model equation was based upon the correlation coefficients and their effect on droplet size. The values were determined from the effects plot (Fig 1), where the coefficient for each significant response was scaled and centred to allow for interpretation. Either a positive or negative effect on droplet size is judged significant if the confidence interval crosses the origin, with insignificant effects excluded from the model, giving the regression model equation:

$$Y_{1}\;=\;31.89\;+\;11.05\;X_{1}\;–\;10.28\;X_{2}\;+\;14.25\;X_{2}^{2}+14.29\;X_{3}^{2}+4.60\;X_{2}X_{3}-10.63\;X_{1}^{2}$$

Where: *Y*_1_ = *Droplet size*, *X*_1_ = *Concentration*, *X*_2_ = *Pressur*, *X*_3_ = *Pump Rate*

The derived regression model equation describes a complex process with linear and/or quadratic relationships for all parameters with droplet size. The most significant factor that showed a linear effect on droplet size is concentration (X_1_), followed by the atomisation pressure (X_2_). When the concentration of Kollicoat IR is increased there is an increase in droplet size; conversely, an increase in atomisation pressure leads to a reduction in droplet size. No significant linear relationship between pump rate and droplet size was seen. All three factors also had a significant quadratic relationship with change in droplet size, with pump rate and atomisation pressure showing very similar values for their coefficients. An interaction between pump rate and pressure (*X*_2_*X*_3_) was also detected, a finding made possible by DOE.

**Fig 2:**
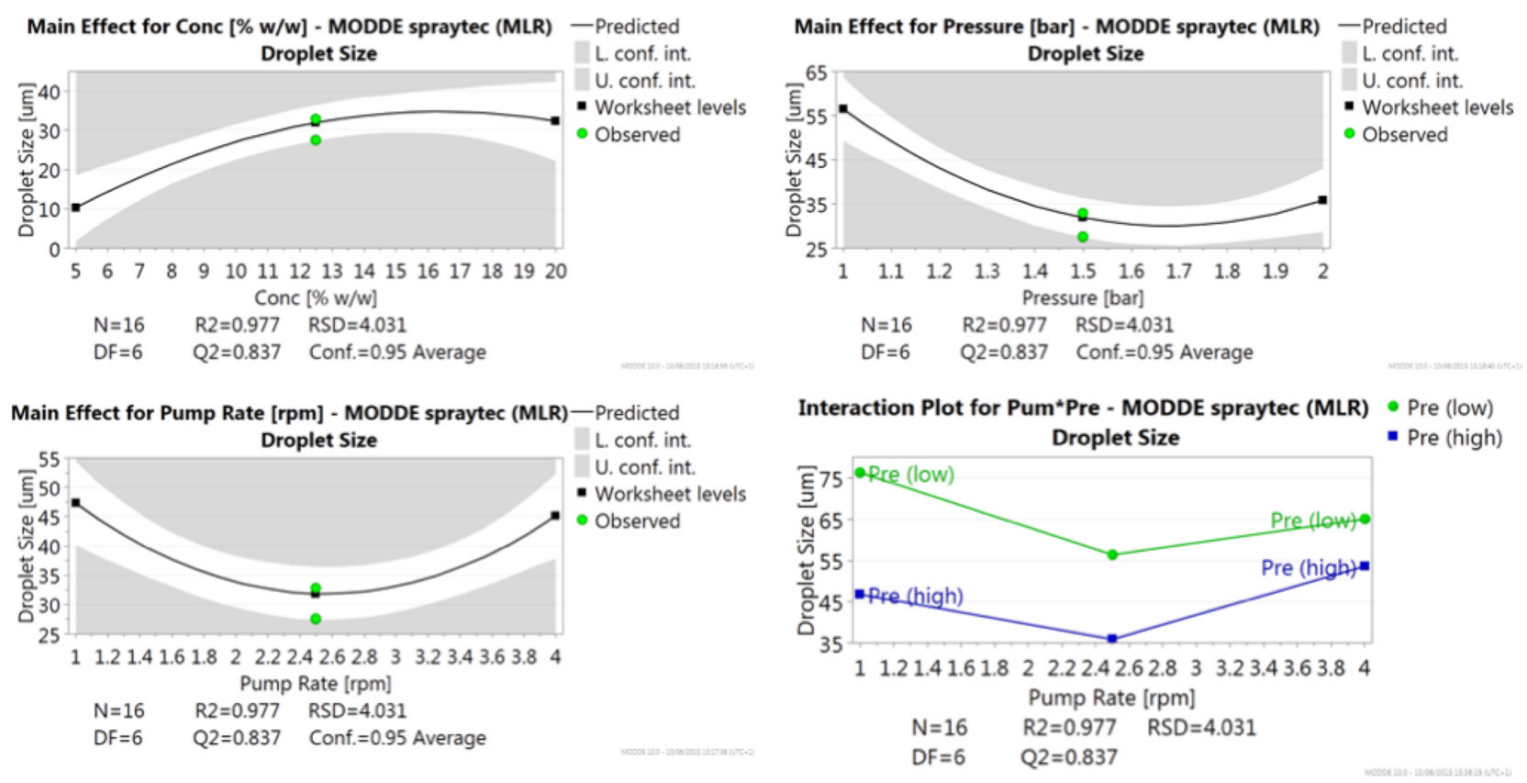
Main Effect Plots for concentration, atomisation pressure and pump rate on droplet size. Bottom right, Interaction Plot for the interaction between pump rate and atomisation pressure. The two lines show atomisation pressure at the low level (1 bar) and high level (2 bar).

More detailed information on the effect that changes in each factor had on droplet size is shown in the Main Effects Plot, Fig 2. The plot for the interaction between pump rate and atomisation pressure is also shown in Fig 2. A clear trend can be seen with an increase in concentration causing an increase in droplet size and an increase in atomisation pressure causing a decrease in droplet size, with the effect of pump rate being more complex. All three plots show a characteristic curved quadratic shape. The increase in droplet size seen with increased Kollicoat IR concentration peaked around the 12.5% centre point, with little change seen at 20%. The relationship seen with increasing pump rate is complex, with the plot forming a clear U shape and the smallest droplets forming approximately between 2 and 3 rpm. The interaction plot between pump rate and atomisation pressure demonstrates finer droplet formation at high pressure. Notably, the difference in droplet size at low and high pump rates is different depending on the atomisation pressure; at low pressure there is a decrease in droplet size from around 77 to 65 μm, whereas at high pressure there is an increase in droplet size from around 47 to 54 μm. This behaviour of a decrease in droplet size with increased flow rate at low pressure and an increase in droplet size with increased flow rate at high pressure is in line with that described for external mix twin-fluid atomisers by Suyari and Lefebvre [40]. They attributed this behaviour to the fact that at low pressure the atomisation equipment operates in a simplex pressure-swirl mode, whereas at high pressure it operates in a simplex-airblast mode. In pressure-swirl mode the increase in liquid flow rate is analogous to an increase in liquid injection pressure; in simplex-airblast mode, due to the high air pressure the increase in flow rate lowers the ALM, thus lessening the atomisation ability. The increase in droplet size seen at the lowest flow rate may similarly be explained by the low flow rate being equivalent to a low liquid injection pressure, resulting in a liquid sheet at the nozzle exit that is more stable and resistant to breakup.

**Fig 3:**
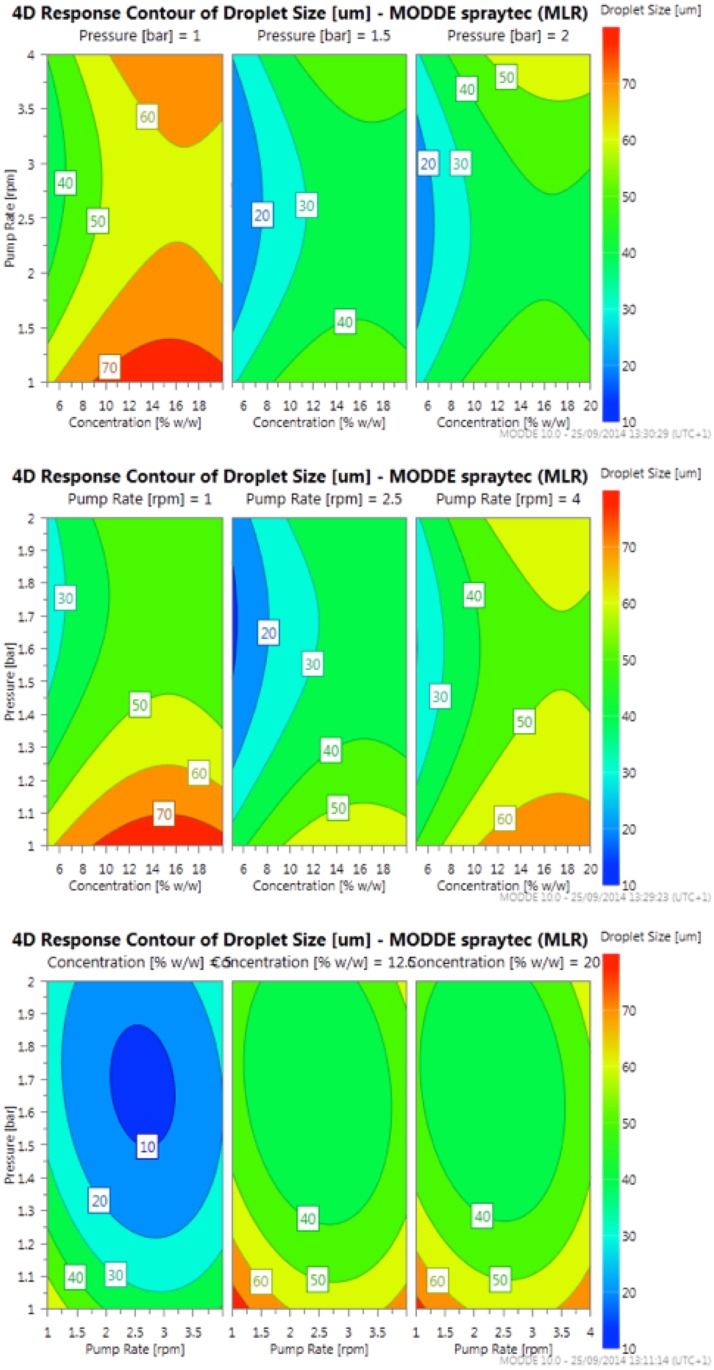
Response contour plot with respect to fixed levels of atomisation pressure, pump rate and polymer concentration.

The response contour plots, Fig 3, give a visual representation of changes in droplet size over the parameter ranges, allowing for optimisation of the process conditions. The plots indicate that in order for very fine droplet formation the major limiting factor is the polymer concentration, since at the mid and high polymer concentrations droplet sizes do not fall below 30 μm unlike at the low polymer concentration.

### Film Coating

The DOE atomisation model allowed for coating of tablets with either large or small droplets. A small droplet VMD of 20 μm and a large droplet VMD of 70 μm were chosen to show the effect of droplet size on the film coat. The conditions to produce droplet sizes as close to these as possible were determined by optimisation of the model using MODDE software. Polymer concentration was set at 8.49% (w/w) (corresponding to a viscosity of 1.73 mPa.s) for both droplet sizes in order for droplet properties to remain consistent, with the exception of VMD. The predicted droplet sizes and the process conditions required to achieve these are shown in Table 2.

**Table 2:** Processing conditions for production of small and large droplets.

Predicted droplet VMD (μm)	Pump rate (rpm)	Atomisation pressure (bar)	Polymer concentration (% w/w)	Coating time (min)
**21.0**	2.56	1.68	8.49	80
**69.3**	1	1	8.49	200

### Film Coat Imaging

Qualitative analysis of the tablet coatings was performed non-invasively using XμCT and CLSM, to examine the effect of droplet size. Processing of the images yielded quantitative information for film coat thickness and porosity, providing a greater comparison between large and small droplet coating quality.

#### Confocal microscopy

**Fig 4:**
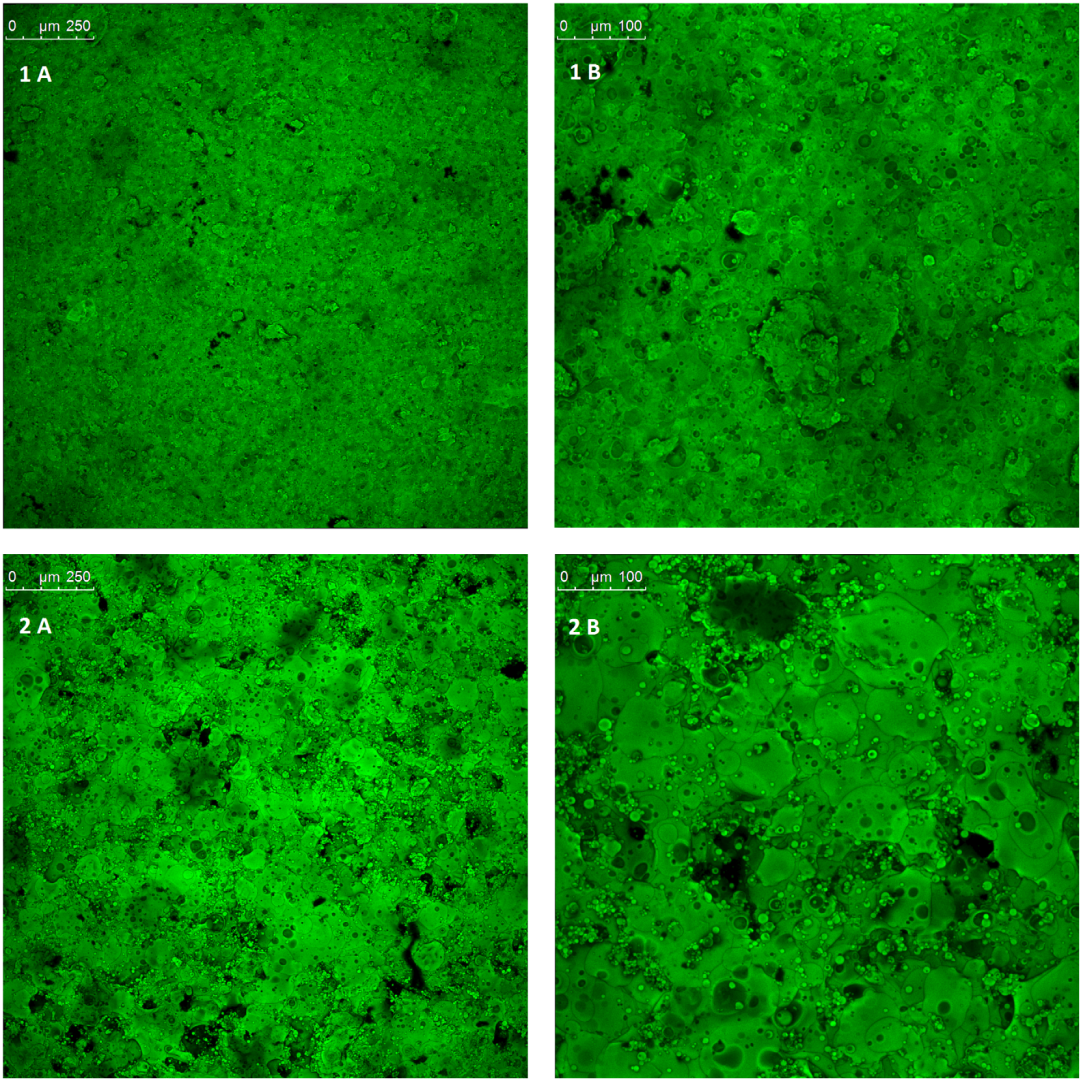
Maximum projection images of the film coat surface, providing a visual representation of surface morphology and film coat uniformity. Aqueous film coat consisting of Kollicoat IR 20% w/w and riboflavin 5’-monophosphate sodium 0.5% w/w as a fluorescent dye. Tablets coated by fluidised bed coating method at defined droplet sizes: 20μm (1) and 70 μm (2). Images were taken at 10x (A) and 25x (B) magnification.

Maximum projection images of the film coated tablets at different droplet sizes are shown in Fig 4. A marked difference can be seen between the two batches. The film coatings of 20 μm droplets are clearly more uniform and complete when compared to the 70 μm droplet coatings. Dark spots in these images indicate areas of low or no coating (pores); the smaller droplet size coated tablets (1 A and 1 B) appear to have a much more complete coating, with fewer dark spots visible when compared to the larger droplet size. Furthermore the smaller droplet coated tablets display a more consistent texture and colour, with the larger droplets forming patches of increased intensity of fluorescence indicating poor homogeneity. Unlike the small droplet coatings, in the large droplet coatings droplet outlines are visible, most apparent in the 25x magnification (2 B). This would suggest a greater water content for the large droplet coats through insufficient water evaporation, however TGA analysis showed no significant difference (P = 0.31) in water content between small and large droplet coatings, with values of 2.68±0.03% and 2.51±0.14% respectively.

**Fig 5:**
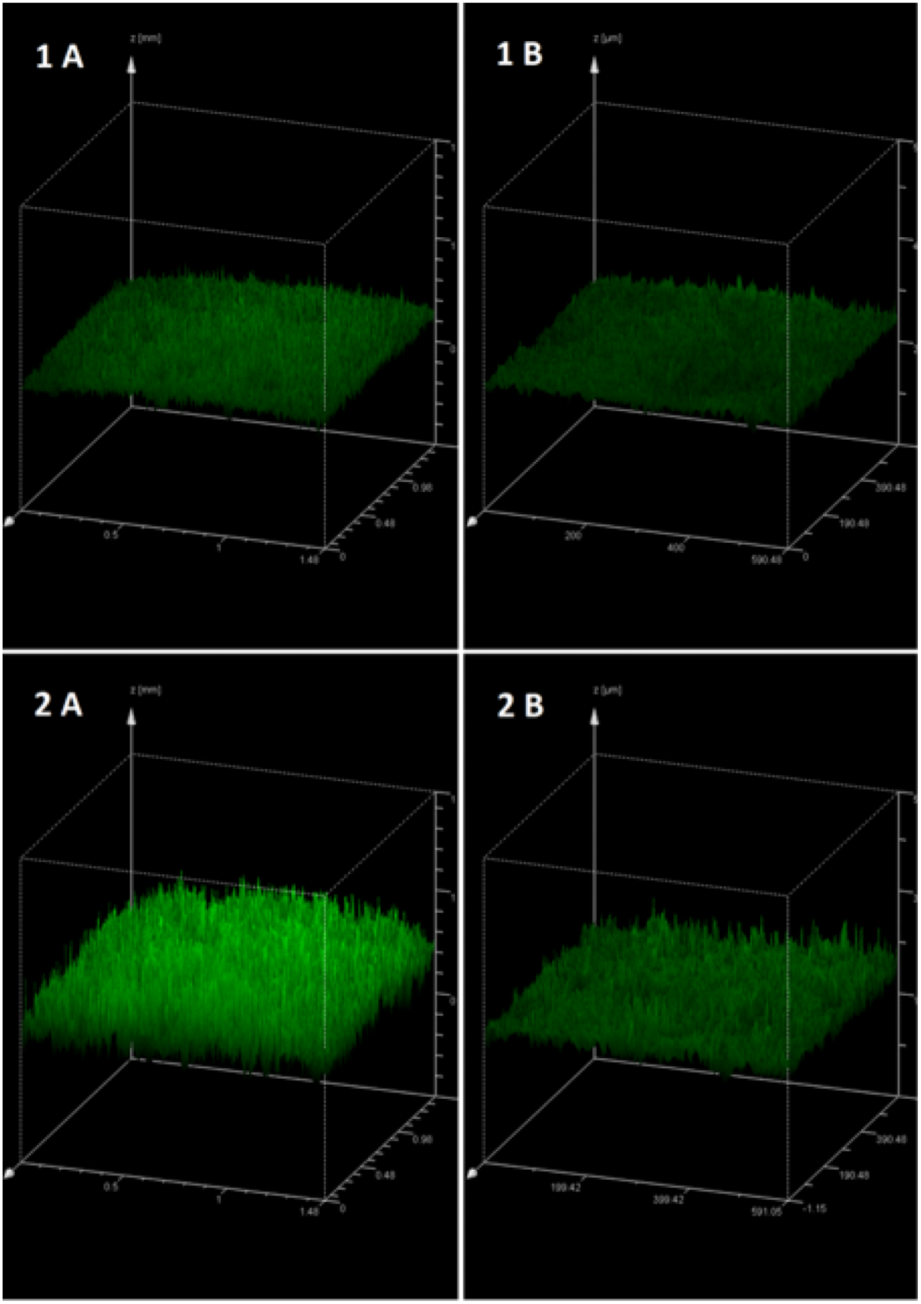
3D CLSM image showing fluorescence intensity at each image layer, providing a visual representation of surface morphology. Film coat consisting of Kollicoat IR 20% w/w and riboflavin 5’-monophosphate sodium 0.5% w/w as a fluorescent dye. Tablets coated by fluidised bed coating method at defined droplet sizes: 20μm (1) and 70 μm (2). Images were taken at 10x (A) and 25x (B) magnification.

**Fig 6:**
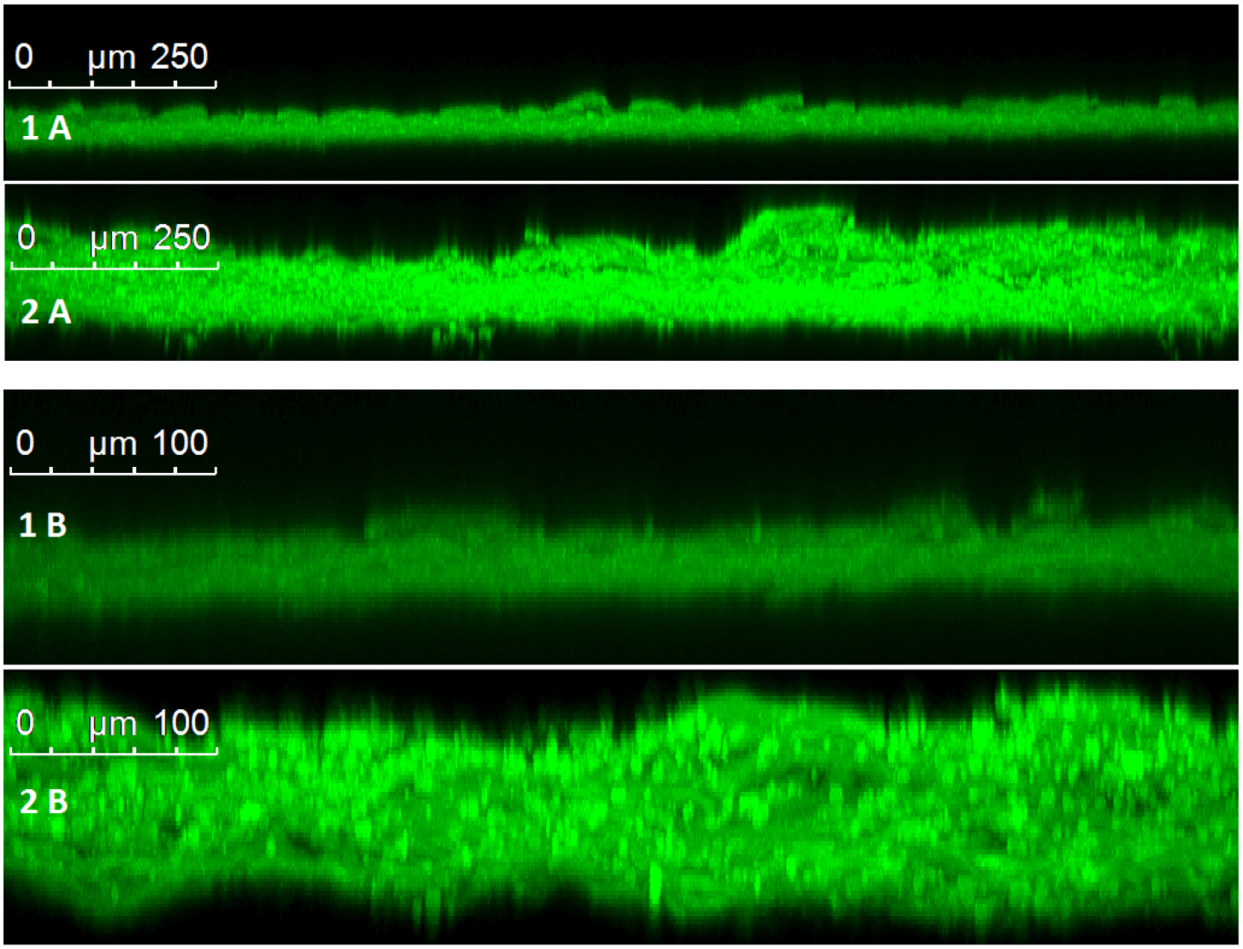
Transverse view of maximum projection images showing the film coat thickness and morphology. Film coat consisting of Kollicoat IR 20% w/w and riboflavin 5’-monophosphate sodium 0.5% w/w as a fluorescent dye. Tablets coated by fluidised bed coating method at defined droplet sizes: 20μm (1) and 70 μm (2). Images were taken at 10x (A) and 25x (B) magnification.

Pixel fluorescence intensity of images at sequential planes was used to generate a 3D projection (Fig 5) of the coating to provide a representation of the surface roughness. These images indicate a thicker coating with large droplets and complement the maximum projection images by showing a rougher surface for the tablets coated with large droplets (2). A transverse view of the maximum projection images can be seen in Fig 6 and gives a non-invasive cross-section of the film coat and the coat-core interface. Since film coat thickness was assumed to be largely dependent on the solid content of the film coat and this was corrected for by coating time, no difference in coating thickness was expected between large and small droplet coatings. Small droplet coatings however are much thinner when compared to the large droplet coatings, with the differences actually being in a similar magnitude as the difference in coating time (2.5x). This may be due to a higher porosity seen with the large droplet coatings.

The images obtained by CLSM suggest that small droplets have produced a thinner, more compact coat that is more homogenous, complete and smooth. This can be attributed to the more efficient evaporation of small droplets due to their greater surface area to volume ratio. These findings are significant since the differences seen between the coats may impact upon the overall tablet properties.

#### Micro-CT

**Fig 7:**
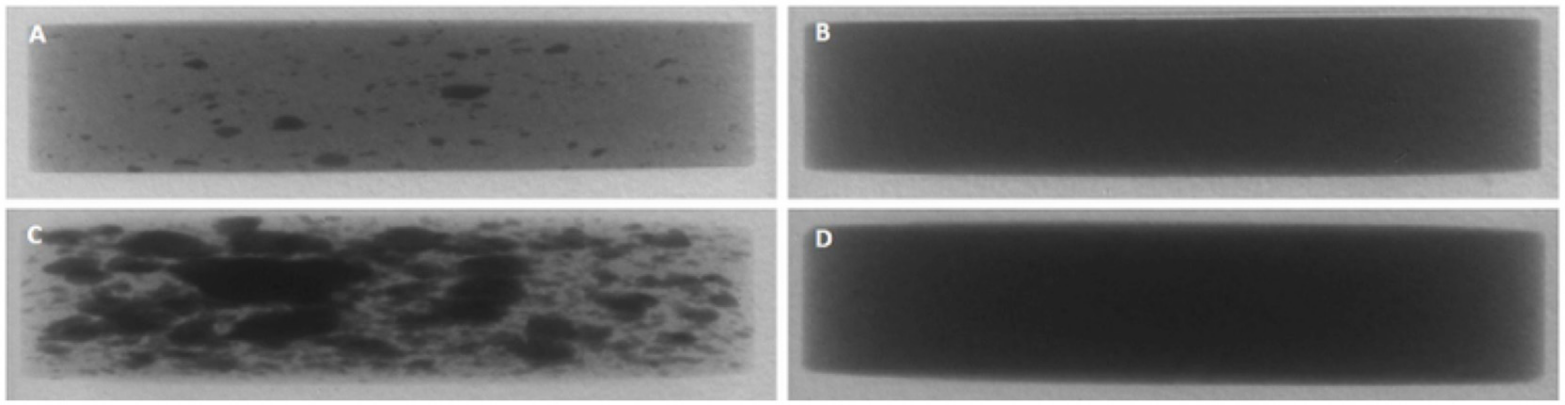
Transverse view of XμCT reconstruction of placebo tablets (13mm) containing contrast material in the core. Contrast material Bi_2_O_3_ is included at 5% w/w and 10% w/w (milled), A and B respectively. BaSO_4_ is included at 10% w/w and 20% w/w (milled), C and D respectively.

**Fig 8:**
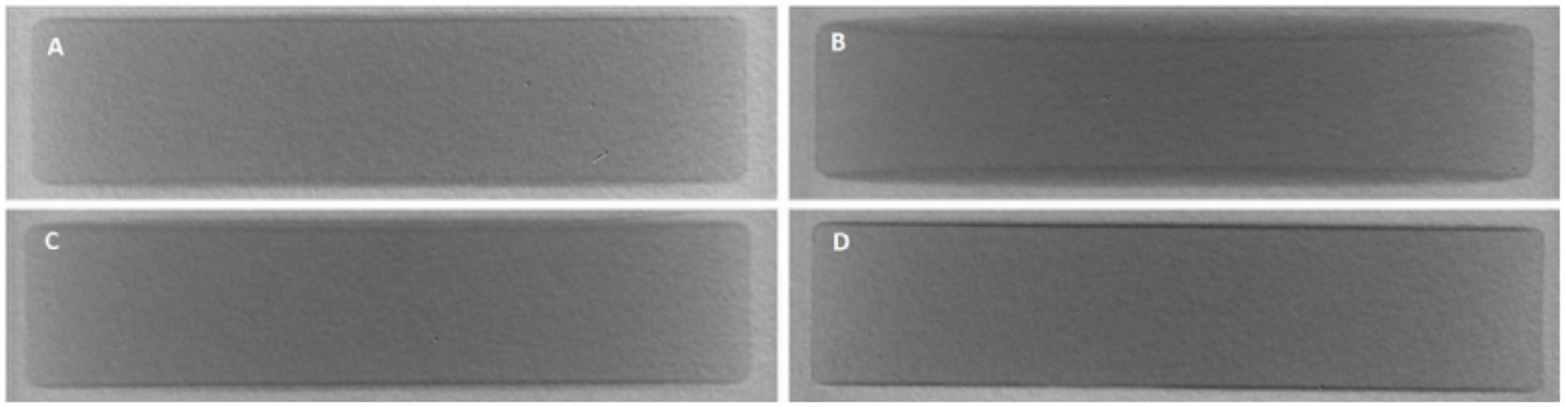
Transverse view of XμCT reconstruction of placebo tablets (13mm) containing contrast material in the coat. Contrast material Bi_2_O_3_ is included at 1% w/w and 2.5% w/w, A and B respectively. BaSO_4_ is included at 2% w/w and 5% w/w, C and D respectively.

XμCT was used to complement confocal data to assess film coat quality and characteristics. Imaging of the tablet core alone was not possible due to low radiopacity shown by the tablet core excipients and Kollicoat IR, as measured using an aluminium step wedge to compare against aluminium standards. Barium sulphate (BaSO_4_) and bismuth(III) oxide (Bi_2_O_3_) were tested as contrast materials for incorporation into both the tablet core and the polymer coating to increase radiopacity. BaSO_4_ has been used extensively in orthopaedic surgery as a radiopacifier ingredient in bone cement to monitor the healing process after fixation of artificial joints [44]. Bi_2_O_3_ is similarly considerably used as a radiopacifier component of dental cement for peri-/postoperative assessment [45]. XμCT reconstructions in Figs 7 and 8 show transverse views of the entire tablets. Addition of either contrast material increased radiopacity enough for successful imaging. The distribution of the contrast material within the tablet core was initially not homogenous, with clumps visible in the reconstructions where contrast material had been blended with the rest of the formulation. Co-processing of the contrast material with the formulation by milling then vastly improved homogeneity of the tablet core. Contrast material inclusion in the coat similarly increased radiopacity for successful imaging. Increasing contrast material concentration in the film coat produced sharper, more defined images, as shown in Fig 8B and 8D.

To determine the effect of droplet size on film coat Bi_2_O_3_ (2.5% w/w) was added to the coating solution. Bi_2_O_3_ was chosen as contrast material since BaSO_4_ demonstrated similar radiopacity but at a higher concentration. The parameters for obtaining the defined droplet sizes caused issues with effective coating with contrast material. At the higher pump rate of 2.56 rpm, Bi_2_O_3_ was readily pumped and atomised. The lower pump rate of 1 rpm necessary for large droplet production proved more challenging and required reduction in the coating solution pumping length, due to the increased transit time of the insoluble bismuth oxide.

**Fig 9:**
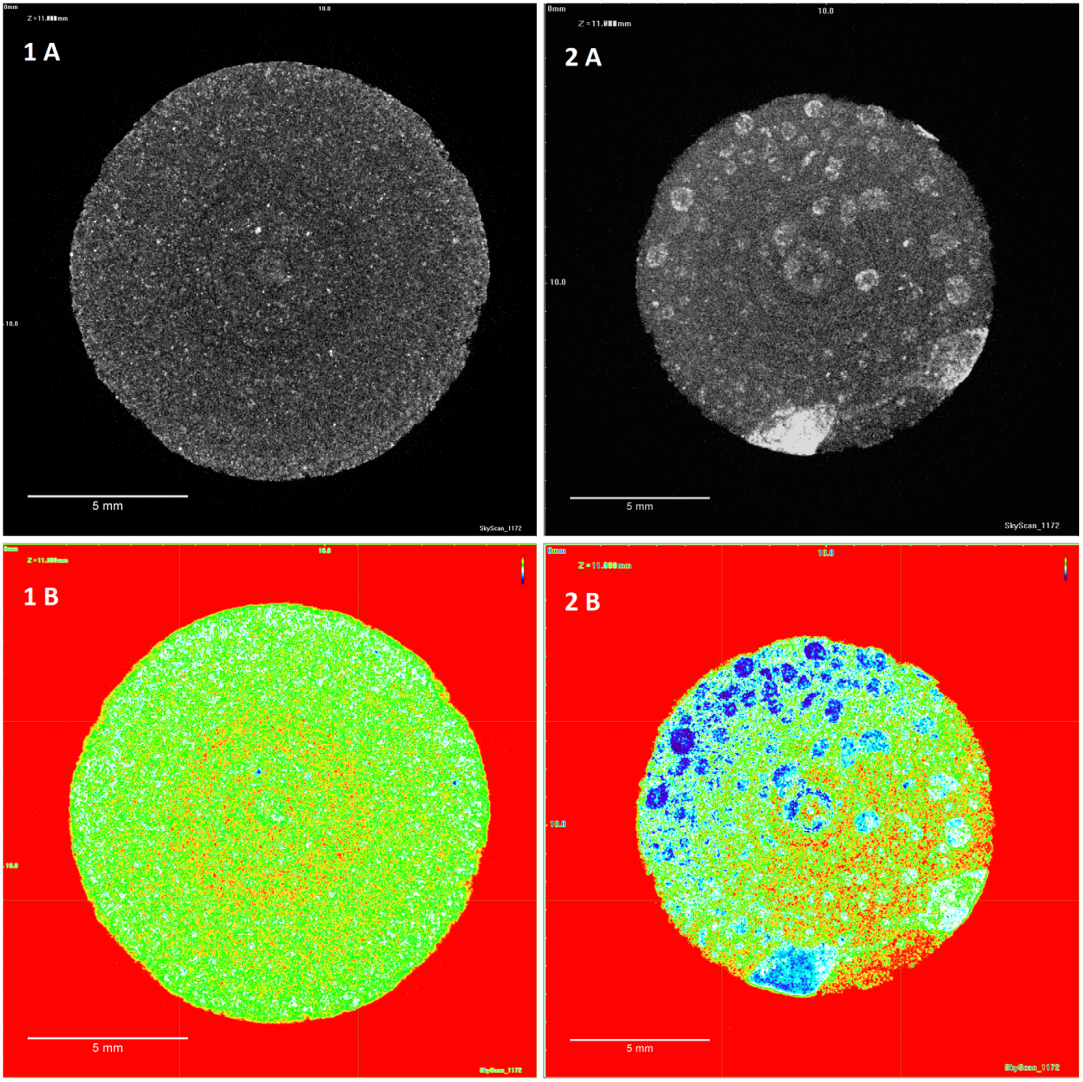
Maximum projection and heat map images of the tablet surface of XμCT reconstructed film coated tablets of either large or small droplet size. Placebo tablets were coated with Kollicoat IR and bismuth oxide (2.5% w/w). Droplet size coatings of 20μm (1) and 70 μm (2) are shown in maximum projections (A) and heat maps (B). Heat maps demonstrate coat uniformity, with blue areas and red areas representing high and low intensity of radiopacity, respectively.

Fig 9 shows the XμCT maximum projections of the top tablet surface, coated with either large or small droplets. The surface images for the small droplet coating complement the confocal data by showing a homogeneous, uniform coating. Similarly, the surface of the large droplet coating shows large droplet artefacts on the coating surface and poor homogeneity. These differences are particularly clear in the heat map images.

#### Film coat thickness and porosity

Analyses of film coat porosity for XμCT reconstructions were performed using two different techniques. Film coat porosity, thickness and roughness analysis was also performed for fluorescent coatings. Porosity measurements were used as an indication of film coat uniformity, with lower porosity representing a more concise coating. The results for the XμCT reconstructions are shown in Table 3. Both techniques for film coat porosity assessment show similar results and similar deviation. Large droplet coated tablets showed approximately double the coating porosity of 44.3±7.1% and 32.8±6.7% compared to the small droplet coating porosity values of 21.4±4.1% and 16.4±3.3% respectively, using each technique.

**Table 3:** Surface porosity measurements of film coatings analysed by XμCT. Porosity has been measured using either the CTAn or ImageJ technique.

Coating Porosity of XμCT Scans by Two Methods			
	Droplet Size	Mean (%)	SD
**CTAn**	Small	21.4	4.1
	Large	44.3	7.1
**ImageJ**	Small	16.4	3.3
	Large	32.8	6.7

The results for the fluorescent coatings, shown in Table 4, show a similar trend, with large droplet coatings having double the porosity of small droplet coatings at 30.0±6.0% and 15.1±3.2% respectively and showing comparable values with the XμCT reconstructions. The film coat thickness measurements show that large droplet coatings are substantially thicker at 114.2±18.1μm compared to small droplet coatings at 48.4±8.1μm, as was visible from the transverse views of the maximum projection images. The greater surface roughness values for the large droplet coated tablets were not deemed significant (P > 0.05).

**Table 4:** Porosity, thickness and roughness measurements of fluorescent coatings. Maximum projection images were analysed using ImageJ. Coatings produced by large and small droplets are compared.

Film Coat Thickness and Porosity Measurements for Fluorescent Coatings						
	Porosity (%)		Thickness (μm)		Roughness—RMS (μm)	
	Mean	SD	Mean	SD	Mean	SD
**Small**	15.1	3.2	48.4	8.1	6.6	2.4
**Large**	30.0	6.0	114.2	18.1	12.8	6.2

## Conclusions

DOE successfully generated a robust model capable of predicting the impact that altering process parameters had on droplet size. All three CPPs under investigation were verified as having a significant impact on droplet size contributing to a complex atomisation process. This approach provided a wealth of information and insight into the process in a short time and allowed for droplet size optimisation that would not have been easily achieved otherwise.

Film coat characterisation by CLSM and XμCT provided complementary qualitative and quantitative information. Small droplets were shown to produce a more complete and concise film coating, and are expected to benefit from enhanced stability as a result of lower porosity and be less at risk to detrimental over-wetting. The increased thickness of large droplet coatings may be as a result of greater porosity of these coatings. The incorporation of a commonly used radiopaque contrast material for XμCT imaging of a film coat was designed to overcome a major limitation of XμCT, that is poor contrast between tablet and coating materials [39]. The wider implications could extend beyond coating, for example with inclusion of contrast materials into tablet cores for non-invasive analysis of internal tablet structure by XμCT. Another application could be to study homogeneity, not only within tablet cores but also powders.
